# Quality of Life as Medicine III. A Qualitative Analysis of the Effect of a Five-Day Intervention with Existential Holistic Group Therapy or a Quality of Life Course as a Modern Rite of Passage

**DOI:** 10.1100/tsw.2004.10

**Published:** 2004-03-04

**Authors:** Søren Ventegodt, Birgitte Clausen, Maja Langhorn, Maximilian Kromann, Niels Jørgen Andersen, Joav Merrick

**Affiliations:** ^1^ The Quality of Life Research Center, Teglgårdstræde 4-8, DK-1452 Copenhagen K, Denmark; ^2^ Vejlby Lokalcenter, Vejlby, Denmark; ^3^ Institute of Anthropology, University of Copenhagen, Denmark; ^4^ Quality of Life Bookstore, Copenhagen, Denmark; ^5^ Norwegian School of Management, Sandvika, Norway; ^6^ National Institute of Child Health and Human Development, Office of the Medical Director, Division for Mental Retardation, Ministry of Social Affairs, Jerusalem and Zusman Child Development Center, Division of Pediatrics and Community Health, Ben Gurion University, Beer-Sheva, Israel

**Keywords:** quality of life, philosophy, QOL, human development, holistic medicine, public health, group therapy, Denmark

## Abstract

Existential group therapy seems to be a very efficient way of inducing the holistic state of healing, described in the holistic process theory of healing. We have designed a series of four quality of life (QOL) and health courses of 5-days duration called “Philosophy of Life that Heals – Courses in QOL and Personal Development”. The four courses are meant to be taken over four consecutive years. They contain training in philosophy of life and existential theory as well as exercises in holding: awareness, respect, care, acknowledgment, and acceptance. The courses teach the participants respect, love, and intimacy; help them to draw on their seemingly unlimited hidden resources; and inspire them to take more responsibility for their own life. Exercises are accomplished with a partner chosen at the course as: (1) a person you like, (2) a person you do not know already, or (3) a person to whom you want to give help, support, and holding more than you want to get help from him or her. Pilot studies with 5-day quality of life interventions that combine training in quality of life philosophy with psychotherapy and bodywork have proved effective on patients with chronic pain and alcoholism. The present design aims to take this a step further and engage the patients in a process of personal growth that will last for years. The aim is to lead them to a stabile state of quality of life, health, and ability, from where they will not again fall into sickness and unhappiness. The focus of these courses is as much on prevention as is it on healing. The existential group therapy induces spontaneous healing of body, mind, and soul that seems to be highly efficient with hopefully lasting results. Every course is intended to give an immediate improvement in the quality of life, so its efficiency can be measured with the square curve paradigm. We have studied the participant’s accounts from their experience with the courses and have analyzed the remarkably large, qualitative changes in the state of being, quality of life, health, and consciousness, which many participants experience during the course. The long-term and preventative effects of the courses have yet to be documented.

### INTRODUCTION

The domain of existential holistic therapy is the whole person, the holos, the soul, the deep self, higher self, real self, or simply the self, in contrast to the ego, false self, or illusory self. Existential psychotherapy[1] addresses the emotional aspect of the human mind related to death, freedom, isolation, and meaninglessness. Existential holistic therapy addresses the state of the persons *wholeness*[2] or "soul". Existential holistic therapy is not existential psychotherapy, as it addresses the body, the feelings, the person's philosophy of life, and the spirit to the same extent as it addresses the psyche, normally translated in psychotherapeutic practice into something like "the emotional mind".

Contrary to existential psychotherapy, existential holistic therapy finds the philosophical, verbalized, and conceptualized part of consciousness very important. Existential holistic therapy is not based on Freud's concept of the sexual urge, but on the concept of the purpose of life, which is embedded in the wholeness of the human being and not in any parts of it[3]. Existential holistic therapy makes an equal point on holding and processing[2] and the belief that healing of the human existence at large improves health, quality of life (QOL), and the ability to function[4,5,6]. Contrary to neo-Freudian (interpersonal) psychodynamic theory[7,8,9], it is assumed in existential holistic therapy that the human being is a product of his or her intentions and particularly of his or her original purpose of life and subsequent denials of this purpose[10,11,12,13].

The holistic process theory of healing is strongly inspired by Jung's theory of the ego, the self, and the shadow[14,15]. Existential holistic therapy can be carried out in an individual[16,17] or group setting[18,19,20]. Whereas existential psychotherapy is rather depressive concerning the fundamental human conditions — life being fundamentally meaningless, everybody being basically isolated, freedom being in essence emptiness, and life as a matter of fact a relentless enterprise ending up with only death — in contrast, existential holistic therapy is positive in each approach.

It looks at life as basically meaningful and coherent. It is true that we are free, but freedom is not emptiness as it is our ticket to autonomy. It makes it possible for us to be our self with our own consciousness and perspective of life and individuality, and most importantly our own personal and unique purpose of life, our personal mission of life[3]. And it is really true that we shall die in the end, but we can die peacefully, happily, and saturated of days, only if we lived our purpose. Life is fundamentally good in existential therapy; love, strength, pleasure, and sexuality are gifts that are the human being's inborn right. To keep up the positive perspective of life is of greatest importance to existential holistic therapy. In accordance with this, many of the participants have experiences with strong positive emotions. These experiences sometimes appear to transform their lives within only a few days of therapy[18,19].

### THE HOLISTIC PROCESS OF HEALING

New understanding of the holistic process of healing makes it possible to intensify and accelerate the therapeutic process. The life mission theory[3,10,11,12,13,14] states that everybody has a purpose of life and happiness comes from living out this purpose and succeeding in expressing the core talent in life. To do this, it is important to develop as a person into what is known as the natural condition, a condition where the person knows himself and uses all his efforts to achieve what is most important for him. The holistic process theory of healing[2,20] and the related quality of life theories[4,5,6] state that the return to the natural state of being is possible, whenever the person gets the resources needed for existential healing. The resources needed are, according to the theory, awareness, respect, care, acknowledgment, and acceptance with support and processing in feeling, understanding, and letting go of negative attitudes and beliefs. The preconditions for holistic healing to take place are trust and the intention that the healing *will* take place. Existential healing is not a local healing of any tissue, but a healing of the wholeness of the person, making him much more resourceful, loving, and knowledgeable of himself and his own needs and wishes. In letting go of negative attitudes and beliefs, the person returns to a more responsible existential position and an improved quality of life. The philosophical change of the person's healing is often a change towards preferring difficult problems and challenges, instead of avoiding difficulties in life[21,22,23,24,25,26,27,28]. The person who becomes happier and more resourceful often also becomes more healthy, more talented, and more able to function[11,18,19].

### THE ROLE OF PHYSICIAN OR THERAPIST

At a course on quality of life, where the purpose is existential healing through the use of existential holistic group therapy, the leader of the session will take the positive perspective of life, which s/he will hold and share with the participants through the course. This is done by using examples from working with the group members one by one, through holding (which gives rich opportunity to demonstrate the nature of attention, respect, care, acknowledgment, and acceptance[1,11,12]), and through processing from the centers of mind, heart, and body[2,18,19,20]. In that way, the therapist will act as the good parent, who enjoys love and admires the participants. If you are accustomed to the "negative attitude" of existentially oriented psychotherapy, holistic therapy is positive to an almost nauseating degree; the focus is on love, good will, friendship, trust, honesty, being there for each other, and quality of life philosophy[21,22,23,24,25,26,27,28], stressing the good nature of man and the wish-fulfilling nature of the universe allowing the hidden resources of the participants to be taken into use.

We do not call the patients "patients", as we prefer to call them "participants" to acknowledge, respect, and actually stress their human integrity and to give them empowerment. To call them patients tends to turn them into the role of a "patient", and thus tends to take power and responsibility away from them.

We find it important to reflect over the feedback that we collect in written form from most of the participants, about a week after the end of the course. This is one way to evaluate the success of the intervention, by analyzing the content and meaning of the core and conclusive statements from the participants.

### EXISTENTIAL HOLISTIC GROUP THERAPY

Existential group therapy seems to be a very efficient way of inducing the holistic state of healing[2,20]. During the last 5 years, a series of four quality of life and health courses have been developed by Søren Ventegodt and nurse Birgitte Clausen. The common title of the courses is "Philosophy of Life that Heals – Courses in QOL and Personal Development". The title corresponds to the books for the courses[16,17], which give a popular introduction to the holistic process theory of healing[2]. The four courses are each

5-day interventions, meant to be taken over four consecutive years. They always start with training in philosophy of life[21,22,23,24,25,26,27,28] and existential theory[3,10,11,12,13] as well as exercises in holding: awareness, respect, care, acknowledgment. and acceptance.

The courses teach the participants respect, love, and intimacy; help them to draw on hidden resources; and inspire them to take more responsibility for their own life. Exercises are accomplished with a partner chosen at the course as: (1) a person you like, (2) a person you do not know already, or (3) a person to whom you want to give help, support, and holding more than you want to get help from.

Pilot studies with 5-day quality of life interventions that combine training in quality of life philosophy with psychotherapy and bodywork have proved effective on patients with chronic pain and alcoholism[18,19]. The concept of these courses is simpler as they focus on the philosophical dimension and holding. Where psychotherapy and bodyworks were guiding the processes in these 5-day interventions, the holistic processes of the courses based on existential group therapy were mostly spontaneous. This seems to be highly efficient, as everybody already seems to know intuitively his or her way back to life, taking into use the self-healing powers of mind, body, and soul. Some of therapeutic tools in use are described elsewhere[2,16,17,20].

The series of courses invites the participants to define a project of personal development, lasting several years. Every course is intended to give an immediate improvement in the quality of life, so its efficiency can be measured with the square curve paradigm[29]. We have studied the participant’s accounts from their experience with the courses and have analyzed the remarkably large, qualitative changes in the state of being, quality of life, health, and consciousness, which many participants experience on the course.

### THE RITE OF PASSAGE

In most premodern cultures, today often believed to be “primitive” — like the native Americans, the black Africans, or the Australian aboriginals — there have always existed powerful rituals that served man in renewing himself and his life by reconnecting to some kind of life force and bringing about a state of healing. An illustrating example of the rite is the sun dance, where the natives dance for four consecutive days and nights without eating or drinking, eventually entering a state of mind where they realize the true values in life.

In our western culture, the transitional rites have been replaced by much less intense traditions such as birthdays, Christmas or other holidays, marriage, confirmation, and students´ binge. Modern society does not have rituals any longer that enable the complete transformation or renewal of man, taking him from one state of being to another.

We have observed that the quality of life courses focusing on personal development and existential healing often take such a form and such an intensity, as to substitute for the classical rite of passage. The collected material indicated that the intensity of experiencing personal transformation and the magnitude of the overall transformation of personality, at least for some of the participants, could be compared to the intensity of the transitional rituals in the primitive cultures.

This article is based on 21 personal accounts from the participation and centered around the personal transformation of the participants and growth during the course and afterwards. Every year, there are some participants who get into personal problems and ethical dilemmas, giving them difficult or even negative experiences in and after the course. The participant with negative results is not the issue for this paper, but it is important to notice that every strong cure can have negative side effects. The precourse assessment is important with the aim to ensure that only people with sufficient personal resources to complete the course are allowed to participate[20].

## A COURSE IN QUALITY OF LIFE

### Philosophy of Life that Heals

The primary purpose of the course is to improve the quality of life of the participants and to get them to know their "self". Health and the ability to function are often also improved, but healing is not the main objective of the course. The improvement of quality of life happens as the participants get to know themselves better and discover their hidden potentials. The first 5-day course is an introduction to personal development, where the participant realizes what he or she could experience in life, but in a better version. The second 5-day course is an effective confrontation with an important and destructive pattern of life. The third course gives the participants training in the "Life Mission Theory"[3], which often gives the participants a breakthrough to a personal acknowledgment of their purpose of life or life mission. The experience is rediscovering who they really are deep down in their soul and existence — the meaning of life.

### The Ritual and Social Space

In order to understand the rite as something else and more than a quality of life course, we can apply Edwin Ardener's interpretation of the rite of passage[30]. Ardener understood the rite as a *synchronicity* of thought, speech, and action in a physical space. This synchronicity converts the space into a "social space", and the rite generates an important meaning for the participants. Symbols related to the ritual can be understood in the context of this meaning. The course held by Ventegodt and Clausen creates such a ritual synchronicity of speech, thought, and action, which transforms the classroom from an accidental physical space and a trivial, academic maneuver to a very special social space, loaded with esoteric meaning.

One participant describes the social space like this: "*The lectures held by Søren, the place itself and all the lovely people, created an atmosphere of great respect and care for each other. (…) We met each other at the bottom of our real being and it is here that all the real alliances between people arise. And I felt like being part of something great…" (Evaluation nr. 17)*[31]. Similar experiences can be seen in almost all the other participant accounts. The social space is described as being characterized by *safety*, *love*, and *respect* and filled with *care*, *holding*, *empathy*, *kindness*, and *personality*.

Another participant: "*Attached to this social space was the creator of scenarios: Doctor Ventegodt. As an object that matched into a synchronicity, uniting action and meaning, he became a symbol that transformed the physical room of the school into the social space. Ventegodt became a symbol for the healthy and good life, and the source for getting there for the participants. Ventegodt is seen as generating the changes and in general, the participants describe him with respect, gratefulness, and enthusiasm."*

Out of the ritual synchronicity of action, speech, and meaning in the course, the ritual social space is created and the participants experience this as something very special. Using the anthropological term "rite of passage", we are able to move a step further and examine the character of the temporary community of the course a little closer and at the same time analyze the nature and inner logic of the experience of the participants, generated in the ritual social space.

### The Course as a Rite of Passage with Separation, Transition, and Incorporation

According to anthropologist Arnold van Gennep[32], the human being undergoes a rite of passage when going from one social, physical, or existential position to another. It marks a change in place, stage, social position, or age. The term "letting go", often used during the quality of life course, describes the moment when the participants gain a cognitive awakening, letting go of the old perspective and thus allowing for the creation of new thoughts and visions to come. The course can therefore be seen as a transformation or a transitional rite, due to the transformation the participants undergo in the courses, when changing their fundamental perspective of life — their philosophy of life.

The transitional rite consists of three stages: *separation, transition,* and *incorporation*. The first stage, *separation,* is when each one of the course participants leaves their normal, usual, and social surroundings behind and takes off for the summer school. The second stage, *transition,* starts during the learning process regarding the theme for the quality of life course. Time is structured around different exercises in order to understand new coherences between body and mind, inner and outer world. When the new understanding of life materializes in a vision of a new life, the transition is made possible. The third stage, *incorporation*, takes place at the end of the course and after the participant is already at home. The participant comes home and is reinserted into his or her normal surroundings, but often something has changed dramatically and they take a new and more interesting role in their own life. Sometimes they are now able to live a life that before was only known to them as a utopia: a dream of a more real, more successful, and more happy life. Ventegodt and Clausen's worldview places the stages of separation, transition, and incorporation side by side as equally important. The course focuses most intensely on the stage of transition.

### The Stage of Transition

The anthropologist Victor Turner has further considerations regarding the rite of passage[33]. He identifies what he calls a "liminal stage". It is an ambivalent condition, as you are neither child nor adult, neither living nor dead. The liminal person, also called the neophyte, cannot be separated from his or her liminal companions, and a community among the neophytes arises centered around this liminality. At the course, the liminal community among the participants is defined, as almost every participant acknowledges having a severe, personal, and existential problem. This fact that all of the participants admit to having existential problems creates an intimate and confidential atmosphere. One participant described his experience of the community: *"Released from our false appearances, that hides the pain in our lives, we were all equal and equally vulnerable. Unprejudiced and vulnerable we created a unique brotherhood of man, where I was able to find peace of mind and harmony, from where I could start developing…" (Evaluation nr. 8)*[31].

Here we see how the social space is important for the individual and his development, and how the holistic treatment is coming about. According to Turner, the typical liminal person finds himself in a situation where he has to be humble and subordinate to the instructors, who instead can help the liminal person to transform from the old position to the new one.

The neophytes are subject to exercises that are a demolition on the one hand and a reconstruction on the other hand. Ventegodt and Clausen, the instructors, have to gain confidence from the neophytes before the neophyte-instructor relationship and the exercises can be accepted. With this confidence, unpleasant elements in the exercises can be accepted. One participant describes the neophyte-instructor relationship like this: *"The way he (Søren Ventegodt) tried to help me, made me feel very safe. He was provocative and hard, but with a good heart. It was the only way for him to make my armament crack down, so I was able to move on…" (Evaluation nr. 2)*[31].

The unpleasant element in the neophyte-instructor relationship is described in general as useful and necessary for the transitional process of development. This underlines the importance of the social relationship between the participant and the instructor, which again generates development for the individual participant. The ritual social space is characterized by the liminal sociality. But what kind of actions and experiences do these social relationships generate?

### Transformation

The anthropologist Colin Turnbull emphasizes that the liminal position seems to bear the characteristics of a *transformation*[34]. The transformation takes place in three central ranges:

1. The first is the change of the participant’s interpretation of reality.
2. The second is the internalization and the use of the instructors worldview through emotional experiences.
3. The third is the change in experiencing past, present, and future coherences and their meaning.

The first change, the transformation of the participants worldview, starts with the participants of all four courses receiving lectures in quality of life theories and models[4,5,6] that express the worldview of the instructors. *“I have understood that man…wants to make a difference in the world, in order to get an increased quality of life. At the same time, exactly this is an obstacle for the things he wants to achieve. This paradox is rooted in the fact that mankind, because of fear and uncertainty, normally do fear change and therefore sticks to the known and safe. But in a respectful and caring environment at the course, new possibilities for personal change and a positive development were created…” (Evaluation nr. 20)*[31].

The participants acknowledge and adapt a new understanding of life in relation to sickness, treatment, faith, salvation, and the good life. The new worldview generates and calls for new ways of action and understanding. New patterns of action are created as the participants practice the exercises during the course. The new worldview and its consequences are experienced. This internalization is a bodily, emotional, and spiritual praxis.

### The Holistic Process of Healing

The transformation of the participant’s life obligatorily starts with *feeling.* Where do the painful gestalts originally come from? Then the participants have to *let go* of the pain and of the negative attitude connected to the traumatic life event, so that in the end they will be able to be themselves and realize their life mission. In order to “let go” of a gestalt, one has to live through the psychological pain that otherwise has been repressed. A participant describes all the things she had to let go of: *“During the course I started to straighten up and look upon which things were important to me in order to conquest the good life. I did let go of the following: “I am scared”, “I am invisible”, “I am not wanted”, “I am alone”, “I am an iceberg”, “it is useless”, “I am a coward”, “I am one big disappointment”, “I am not welcome”, “I am a misfit”, “I am refused”, “I am unhappy”, “I am cold”, “I refuse others”, “I have to do that”… In addition to that, I have learned to take responsibility for my life.” (Evaluation nr. 16)*[31].

In the process of “letting go”, the transformation consists of the participant’s new way of acting and being in his/her life. The process is described as something supranormal, something very special. The experience of transformation is often something magical or spiritual, and often it becomes the symbol for the central change for the participant. Some of the participants talked about a spiritual breakthrough, others of a religious experience, and others again about an existential awakening. For the majority, the transformation had the last character. A participant described that change as: *“Now I see my future in a totally different perspective. I am being optimistic, I am looking forward…What is going to happen in my life, I simply do not know, but one thing is certain: IT WILL NEVER AGAIN BE AS IT ONCE WAS.” (Evaluation nr. 6)*[31].

Almost all the participants expressed that their being in the world had changed since the “letting go” with the change described as an existential, spiritual, or even religious character. The third sign of transformation is when the participants experience a sudden change in the understanding of their past, present, and future. During the quality of life course, the participants have to *realize* the cause of the gestalts in order to let go of them. The past life events are understood in relation to the present problems in a complete new way. For example, one participant, working with regression in a time-line exercise, found a meaningful cause to most of his present day problems, in traumas from his early, fetal life: *"The things that happened to me in my embryonic stage has [I realize now] followed me ever since. I have often felt very lonely and mad. I have often said, that I might as well die, because there was nothing that life could offer me. Another thing that has followed me ever since birth is my confusion." (Evaluation nr. 1)*[31].

Other participants found the roots of their problems as human beings: bad relationship to their parents, incest, violence, alcohol abuse, a life without love, a life with no meaning into it, expectations, jealousy, and so on. In this way, the experiences of the participants were transformed, also their personal history, reality, and now they have a starting point for future experiences.

### DISCUSSION

The qualitative analysis in this paper was based on written reports. It might have been of value to interview the participants to get a picture with more depth and detail. The interpretation was done in accordance with the scheme of the quoted authors conception of the rite of passage. It could have been of value to use a quantitative method of evaluation also; measuring with a short quality of life questionnaire like QOL5[35] and using the square curve paradigm[29] is the next natural step in assessing the overall value of the course for the participants.

The value of the study is limited to the present period, as we do not know how the participants will develop in the future; a study with a control group following the participants over some years would give a more precise picture of the objective, therapeutic value gained by the course.

When this is said, the qualitative approach has some advantages compared to the quantitative; deep existential transformations are not likely to be well pictured in any of the existent quality of life questionnaires. We have chosen this method for this study as we believe that the quality and extent of the participant's experiences of personal transformation, shift of life perspective, and development of their philosophy of life, which was the prime purpose of the quality of life intervention, are better illustrated by a few well chosen words from the participants than by series of numbers.

### CONCLUSIONS

When the course is finished, the participants have to deal with a new challenge to implement what they have learned about themselves and their life into their normal surroundings. This can cause serious problems, since many people face difficulties with their network when they change. The participants become more self-conscious, more driven from within, and less superficial. Serious showdowns with partners, friends, family, and colleagues concerning values and way of life are very common. This is caused by the many factors that restrain people and their freedom to realize themselves along with others, from legislation to all kinds of conventions we are brought up to obey in our society.

It is here that utopia comes into light. Utopia is life-dream that is thought of as an impossible dream to realize, until the moment comes and you succeed in realizing it. The quality of life rite of passage gives the impression that the social utopia, founded on love and friendship, is possible and potential. The ideal is that the citizen in our society can flourish and thrive in his or her own creative self-actualization. The participants that succeed in improving their quality of life, health, and ability to function, seem to be those who succeed in realizing their inner and often secretly and suppressed dreams about love, friendship, and personal development in personal and work life. It is those who fight and succeed in realizing their own personal social utopia. Everything is done to optimize yourself, being good to yourself, and thereby maintaining your quality of life. Like in the primitive cultures, a deep and lasting relationship often arises between the travel partners of the quality of life course — the modern rite of passage ties the participants together in love and friendship for many years to come.

Holistic healing has been a subject for many alternative approaches and until now has not been taken too seriously in the medical community that focuses mostly on biomedicine for the renewal of our medical practice. We believe that it is time to make such approaches a subject for medical science and research[36]. Although the present study is only a small beginning, and it is hard to make conclusions from it, it points to an important new direction that might benefit many patients with all kinds of existential disturbances and problems. Even patients with physical and mental problems may be helped in some cases by obtaining a new and deeper understanding of life, and its purpose and meaning for them.

### ACKNOWLEDGMENTS

The quality of life study was supported by grants from The 1991 Pharmacy Foundation, as well as by supplementary grants from Goodwill-fonden, the JL-Foundation, E. Danielsen and Wife's Foundation, Emmerick Meyer's Trust, the Frimodt-Heineken Foundation, the Hede Nielsen Family Foundation, Petrus Andersens Fond, Wholesaler C.P. Frederiksens Study Trust, Else & Mogens Wedell-Wedellsborg's Foundation, and IMK Almene Fond. The quality of life research was approved by the Copenhagen Scientific Ethical Committee under number (KF)V.100.2123/91.

### BIOSKETCHES

**Søren Ventegodt, MD,** is the Director of the Quality of Life Research Center in Copenhagen, Denmark. He is also responsible for a Research Clinic for Holistic Medicine in Copenhagen and is a popular speaker throughout Scandinavia. He has published numerous scientific or popular articles and a number of books on holistic medicine, quality of life, and quality of working life. His most important scientific contributions are the comprehensive SEQOL questionnaire, the very short QoL5 questionnaire, the integrated QOL theory, the holistic process theory, the life mission theory, and the Danish Quality of Life Research Survey, 1991–94 in cooperation with the University Hospital of Copenhagen and the late pediatric professor Bengt Zachau-Christiansen. E-mail: ventegodt@livskvalitet.org. Website: www.livskvalitet.org

**Birgitte Clausen, RN,** is leader of a secluded ward for people with dementia and dementia coordinator and consultant in Aarhus, Denmark with more than 20 years experience in adult psychiatry and dementia. During the last 4 years, she has worked with Søren Ventegodt on the "QOL as medicine project" and developing existential holistic group therapy. Through her articles and lectures, she has disseminated “The New Culture of Dementia” in both Denmark and Japan. She has participated in the production of several videos in order to inspire relatives, medical staff, and volunteers to understand people with dementia. This project has been granted financial support from the Ministry of Social Affairs in Denmark. She has conducted the first Danish investigation on the effect of sufficient daylight on the human spirit, activity level, and biological rhythm. E-mail: villahvide@mail.dk

**Maja Langhorn Jensen, BA and MA-student** at the Institute of Anthropology, University of Copenhagen. She did her BA-essay on the health understanding and healing practice of Søren Ventegodt. Her primary anthropological interest lies within medical anthropology, focusing on the connection between cultural meaningfulness and physical health. She is currently working as a student assistant on a rehabilitation project at the Danish Cancer Society (Kræftens Bekæmpelse). E-mail: majasverden@email.dk

**Maximilian Kromann, Cand. Phil in Philosophy.** His main field is existential philosophy and studies this field extensively. His most important scientific contributions are the understanding of the nature of man and the preconditions for a successful self-expression. He is also the Director of the Quality of Life Bookstore in Copenhagen, Denmark, where he is coaching patients and customers in selecting literature that can develop their personal philosophy of life. He is also a popular speaker on this topic. E-mail: LivskvalitetsBogladen@livskvalitet.org

**Niels Jørgen Andersen, MSc,** Professor, Department of Innovation and Economic Organization, Norwegian School of Management. This department conducts research and provides teaching in central topics related to innovation, business development, management of global companies, business history, and economic organization. Research activities within the Department are related to four core subjects within the discipline: business history, cooperative organizations, business development and entrepreneurship, and finally studies of industries with a special focus on the electricity industry. He is also the dynamic chairman of the nonprofit organization Stiftelsen Holistisk Medisin Scandinavia that aims to support the scientific development, research, and documentation of complementary and holistic medicine in Scandinavia. E-mail: niels.j.andersen@bi.no. Website: www.bi.no/users/fgl93013/

**Joav Merrick, MD, DMSc,** is Professor of Child Health and Human Development affiliated with the Zusman Child Development Center, Division of Pediatrics and Community Health at the Ben Gurion University, Beer-Sheva, Israel; the Medical Director of the Division for Mental Retardation, Ministry of Social Affairs, Jerusalem; and the Founder and Director of the National Institute of Child Health and Human Development. He has numerous publications in the field of child and human development, rehabilitation, intellectual disability, disability, health, welfare, abuse, advocacy, quality of life, and prevention. Dr. Merrick received the Peter Sabroe Child Award for outstanding work on behalf of Danish Children in 1985 and the International LEGO-Prize (“The Children’s Nobel Prize”) for an extraordinary contribution towards improvement in child welfare and well being in 1987. E-mail: jmerrick@internet-zahav.net. Website: www.nichd-israel.com
